# Ameliorative Effects of Curculigoside from *Curculigo orchioides* Gaertn on Learning and Memory in Aged Rats

**DOI:** 10.3390/molecules170910108

**Published:** 2012-08-24

**Authors:** Xiu-Ying Wu, Jian-Zhong Li, Jian-Zheng Guo, Bao-Yuan Hou

**Affiliations:** Department of Geraeology, First Affiliated Hospital of Suzhou University, Suzhou 215006, China

**Keywords:** curculigoside, *Curculigo orchioides*, Alzheimer’s disease, behaviour study, ameliorative effects on learning and memory

## Abstract

This study was designed to evaluate the ameliorating effects of curculigoside from *Curculigo orchioides* Gaertn on learning and memory in aged rats. In the present study, the ameliorating effects of curculigoside were determined through animal behaviour studies (including step-down test and Y-maze test), and the possible mechanisms were explored by evaluation of the activity of acetylcholinesterase (AchE) and determination of the expression of BACE1. Oral adminstration of the curculigoside (20, 40 mg/kg/day) for 14 days can significantly improve the latency and number of errors in aged rats based on the behaviour study results. In addition, the activity of AchE can be decreased by treatment of the curculigoside (10, 20, 40 mg/kg/day). Moreover, the expression of BACE1 can be down-regulated in the hippocampus of aged rats treated with curculigoside. The results of our present work have indicated that curculigoside can improve cognitive function in aged animals, possibly by decreasing the activity of AchE in the cerebra and inhibiting the expression of BACE1 in the hippocampus. In conclusion, our results suggested that curculigoside can be possible developed as a new drug for the treatment of Alzheimer’s disease in the future.

## 1. Introduction

Alzheimer’s disease (AD), which has very high prevalence in aging people, is characterized by progressive cognitive dysfunction due to the presence of senile plaques and neurofibrillary tangles in the brain regions [1,2]. One of the pathological characteristics of AD is the progressive deposition of insoluble amyloid β protein (A β) as a form of senile plaques [3,4]. In addition, studies have demonstrated that abnormal metabolism of β-amloid precursor protein (APP) is critical for AD pathogenesis [4,5].

AD is one of the leading causes of death in people aged 65 and older, and deaths attributable to AD have been rising dramatically [6]. Therefore, how to alleviate AD has become a serious and urgent problem. However, drugs for treatment of AD are limited because the effects of present drugs are not very good and/or they have severe side-effects.

**Figure 1:**
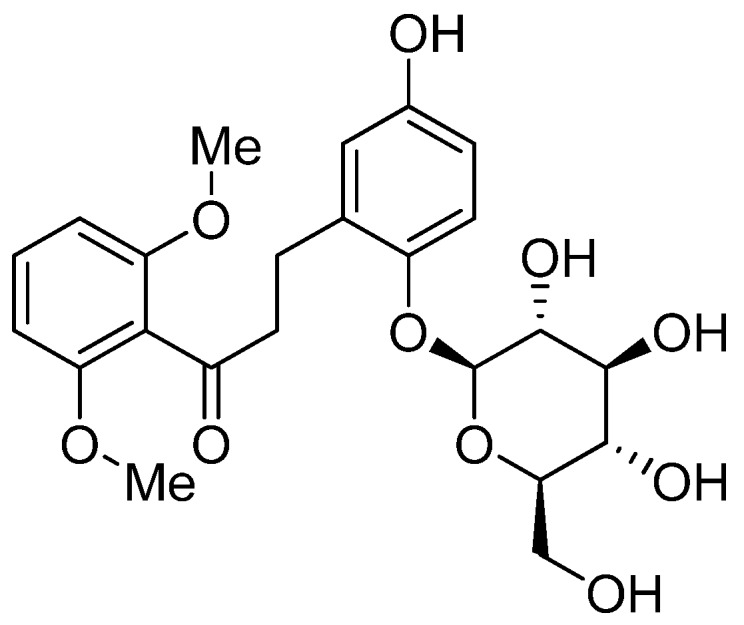
Structure of curculigoside.

For more than a millennium, herbal remedies have been used in Asian countries, apparently safely and effectively, to prevent and alleviate a wide variety of diseases [7,8]. *Curculigo orchioides* Gaertn, a small herbal plant belonging to the family Amaryllidacea, is widely distributed in China, India, Sri Lanka and Japan. It has been traditionally used in folk medicine as the tonic, alterative, demulcent, diuretic and restorative [9,10,11]. Curculigoside, a phenolic glycoside, is the major bioactive compound present in *C. orchioides* (Figure 1). Curculigoside has a wide spectrum of pharmacological activities as an anti-immunostimulant [12], anti-oxidant [13,14], anti-ischemia injury agent [15], *etc.* However, thus far there have been no reports on the ameliorative activity of curculigoside on learning and memory and its possible mechanisms of action. In our present work, a large quantity of curculigoside has been isolated from *C. orchioides*. Then, we studied the ameliorative activity of curculigoside on learning and memory and found in preliminary experiments that it had powerful ameliorative activity. During the present study, we further investigated the ameliorative activity of curculigoside on rats with a model of AD induced by scopolamine so as to elucidate the ameliorative activity and mechanism of this compound, and provide a scientific basis for the clinical use of curculigoside.

## 2. Results and Discussion

### 2.1. Effects of Curculigoside on Learning Performances in Aged Rats in the Step-Down Test

**Figure 2:**
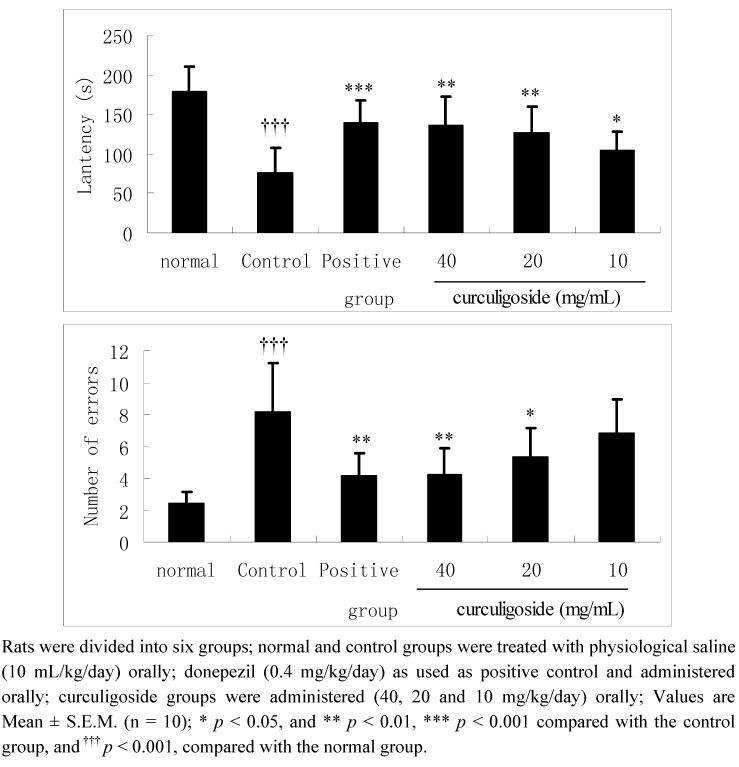
The effect of curculigoside on learning performances in step-down tests.

As can be seen from the Figure 2, in the control group, the latencies were significantly shortened and the number of errors markedly increased compared with the normal group in the step-down test (*p* < 0.001). In contrast, in the aged rats treated with curculigoside (40, 20 and 10 mg/kg/day) for 14 days, the latencies were significantly increased compared with the control rats (*p* < 0.01, *p* < 0.01 and *p* < 0.05, respectively). In addition, the errors number of rats treated with curculigoside (40 and 20 mg/kg/day) for 14 days were significantly decreased compared with the control group (*p* < 0.01 and *p* < 0.05, respectively).

### 2.2. Effects of Curculigoside on Learning Performances in Aged Rats in the Y-Maze Test

**Figure 3:**
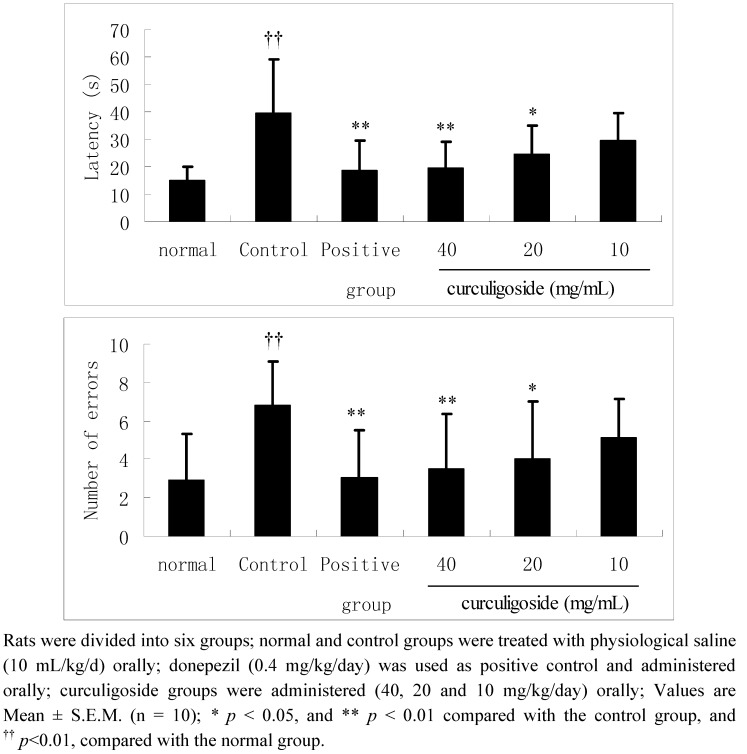
The effect of curculigoside on learning performances in Y-maze tests.

As shown in Figure 3, in the control group, the latencies were markedly prolonged and the number of errors significantly increased compared with normal rats in the Y-maze test (*p* < 0.01). In contrast, after administration of curculigoside (40 and 20 mg/kg/day) for 14 days, the learning performances of aged rats were significantly improved compared with the control group, except in the lower dose group (10 mg/kg/day).

### 2.3. Effects of Curculigoside on Cerebral Acetylcholinesterase Activity

**Figure 4:**
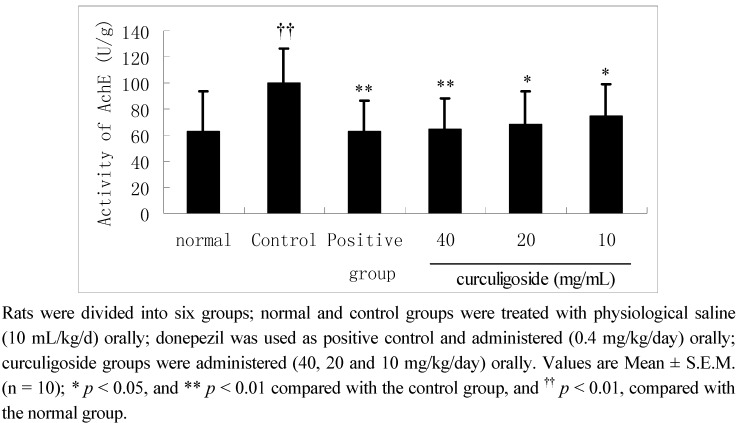
The effect of curculigoside on cerebral acetylcholinesterase activity in aged rats.

To determine the effects of curculigoside on acetylcholinesterase (AchE), the cerebral AchE activity was evaluated and the results were presented in Figure 4. Significant increase of AchE activity was observed in the control group compared with the normal group (*p* < 0.01). However, this increase in the activity of AchE use was reversed when the rats were treated with curculigoside (40, 20 and 10 mg/kg/day) for 14 days compared with the control group (*p* < 0.01, *p* < 0.05 and *p* < 0.05, respectively).

### 2.4. Effect of Curculigoside on Expressions of BACE1

**Figure 5:**
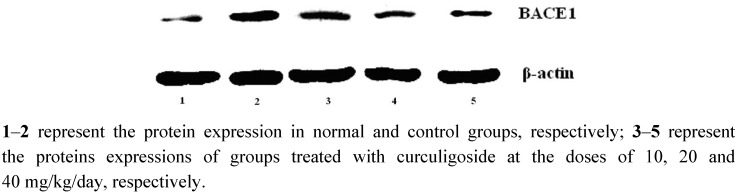
The effect of curculigoside on expression of BACE1 in aged rats.

As can be seen from Figure 5, expressions of BACE1 in hippocampus of control group were up-regulated compared with normal rats; however, the aged rats treated with curculigoside were significantly down-regulated compared with control group.

### 2.5. Discussion

AD is the most common cause of dementia and is characterized by a gradually worsening difficulty in remembering new information [6,16]. Animal behaviour studies are one of the most reliable determinants of animal intelligence, and animal models have been used extensively in the search for novel therapeutic methods for treating AD. [17,18,19]. The step down test, belongs to the passive aviodance response experiments, is developed for the study of learning and memory based on the measurement of step-down latency in passive avoidance and the error numbers [19]. The step-down test and Y-maze test in rats are preliminary and simple models for searching for potential drugs with ameliorative activity on learning and memory. The present study evaluated the ameliorative effects of curculigoside on learning and memory in aged rats using the step-down and Y-maze test. In addition, the results of our present study demonstrated the curculigoside can significantly improve the learning performances in aged rats as evidenced by an increased latency and a decreased number of errors in the step-down test and Y-maze test compared with the control group, which indicated that curculigoside might be useful for the treatment of AD.

Learning and memory are the important functions of our brain, which are related with complex neurophysiologic and neurochemical changes, especially acetylcholine (Ach) levels [20]. Ach has been associated to attentional processes and plays an important effect in cognitive processing [21]. Moreover, the deficiency of Ach is one of the important factors of the AD, and AD can be reversed by preventing the breakdown of Ach in the synaptic cleft [22,23]. From the results of our present work, we can demonstrate that the activity of AChE was significantly decreased in the cerebra of aged rats by treatment of curculigoside.

Aβ deposits are one of the pathological features of AD, and Aβ is the major component of senile plaques in the brain tissue of AD patients [24]. Aβ is derived through sequential proteolytic processing of the APP by β-secretase and γ-secretase, and APP can be cleaved by α-secretase with the Aβ domain [25]. The β-secretase also known as β-site APP cleaving enzyme (BACE1), and several lines of evidence demonstrate that BACE1 inhibitors represent an attractive Aβ-lowering strategy for treatment of AD [25,26]. In addition, from the results of Roberds *et al.* [27], BACE1 is the primary β-secretase activity in brain, and loss of β-secretase activity produces no profound phenotypic defects with a concomitant reduction in β-amyloid peptide, which clearly indicate that BACE1 is an excellent therapeutic target for treatment of AD. In the results of our present investigation, the BACE1 expressions of aged rats treated with curculigoside were significantly down-regulated compared with control group, which indicated that curculigoside may be a potent potential BACE1 inhibitor.

## 3. Experimental

### 3.1. Plant Material

*C. orchioides* was purchased from Tong-ren-tang Pharmaceutical Group and identified as the rhizoma of *C. orchioides* by the department of Traditional Chinese Medicine in our hospital. A vouncher specimen of *C. orchioides* (S20100816–06#) was deposited at our hospital.

### 3.2. Animals

The animals were obtained from the Shanghai Laboratory Animal Center (Shanghai, China). Young and aged male Sprague-Dawley rats (200 ± 20 g, 3 months of age, and 600 ± 50 g, 24–25 months of age, respectively) were used in our study. Rats were kept on a 12 h light/dark cycle with free access to standard laboratory chow and water. Humidity was maintained at 50% and the temperature at 25 °C. Each animal was used only once in the experiment. The experimental protocols were approved by the Animal Care and Use Committee of our hospital. 

### 3.3. Sample Preparation

The following reagents and drugs were used: MeOH (AR), petroleum ether (AR), ethyl acetate (AR), *n*-butanol (AR) were purchased from Sinopharm Chemical Reagent Co., Ltd. (Shanghai, China); silica-gel (100–200, 200–300 mesh) was purchased from Qingdao Haiyang Chemical Co., Ltd. (Qingdao, China), Sephadex LH-20 was purchased from GE Healthcare Co. (Skokie, IL, USA). Scopolamine and donepezil were purchased from Aladdin Reagent Co. Ltd. (Shanghai, China). Rat acetylcholinesterase, AChE ELISA Kit was purchased from Shanghai Boyao Technology (Shanghai, China).

### 3.4. Drugs and Chemicals

The dried and powdered rhizome of *C. orchioides* (40 kg) was extracted under reflux three times (each extraction period lasted 2 h) with 75% aqueous ethanol solution. The solution was concentrated and partitioned with solvents starting with petroleum ether, ethyl acetate, and *n*-butanol. The ethyl acetate fraction was also concentrated under reduced pressure to obtain a residue (1,273.6 g). This ethyl acetate fraction (1,200 g) was eluted through silica-gel (100–200 mesh) with petroleum ether-acetone (20:1, 15:1, 10: 1, 5:1, 2:1, 1:1, 1:5) to obtain a number of sub-fractions A–G. By using a series of chromatographic techniques, such as silica gel column chromatography (200–300 mesh) and Sephadex LH-20 chromatography, curculigoside (2.3 g) was isolated from fraction C.

### 3.5. Analysis of Curculigoside

The isolated chemical compound was identified by ^1^H-NMR and ^13^C-NMR as curculigoside [28]. The spectral data and chemical structure of the chemical compound are as follows:

*White needle crystals*; ESI-MS *m/z*: 489 [M+Na]^+^, 465 [M−H]^−^, it showed the molecular ion at *m/z* 466, which was in agreement with the molecular formula C_21_H_24_O_11_. ^1^H-NMR (600 MHz, DMSO-*d_6_*) δ: 6.98 (1H, d, *J =* 9.0 Hz, H-3), 6.65 (1H, dd, *J =* 3.0, 8.6 Hz, H-4), 9.06 (1H, s, 5-OH), 6.81 (1H, d, *J =* 3.0 Hz, H-6), 5.33 (2H, s, H-7), 3.77 (3H, brs, 2' and 6'-OCH_3_), 6.74 (2H, d, *J =* 8.5 Hz, H-3' and H-5'), 7.38 (1H, t, *J =* 8.5 Hz, H-4'), 4.62 (1H, d, *J =* 7.5 Hz, H-1"); ^13^C-NMR (150 MHz, DMSO-*d_6_*) δ: 127.7 (C-1), 147.5 (C-2), 117.3 (C-3), 114.9 (C-4), 152.4 (C-5), 114.3 (C-6), 61.4 (C-7), 104.5 (C-1'), 156.8 (C-2' and C-6'), 113.1 (C-3' and C-5'), 131.4 (C-4'), 166.0 (C-7'), 56.3 (2' and 6'-OCH3), 102.8 (C-1"), 73.5 (C-2"), 77.2 (C-3"), 70.0 (C-4"), 76.8 (C-5"), 60.8 (C-6").

### 3.6. Protocols

Ameliorative activity of curculigoside on learning and memory in aged rats was evaluated on the step-down test and Y-maze test animal models. The mechanism of curculigoside was explored through evaluating the activity of AchE in brain, and Western-blots were used to determine the BACE1 expression in hippocampal tissues. Dosage of the positive control was determined on the basis of the principle of pharmacokinetics and clinical use. Curculigoside was administered orally, and the dose selection of 10, 20, and 40 mg/kg/day was based on the results of preliminary experiments. Normal and control groups were treated with an equivalent volume of the vehicle (0.5% CMC-Na) that had been used to dilute this chemical compound. 

### 3.7. Preparation of AD Model Rats and Grouping

In our present experiment, scopolamine (1 mg/kg/day, i.p.) was administered 30 min before the training trial to induce memory acquisition impairment in rat [17]. In the behavioural tests (step-down test and Y-maze test) and the AchE activity test, 50 aged rats were randomly divided into five groups (n = 10): three curculigoside tretment groups (10, 20 and 40 mg/kg/day), one control group and one positive control group; another 10 young rats served as the normal group. In the Western-blot assay for BACE1 expression, 40 aged rats were randomly divided into four groups (n = 10): three curculigoside tretment groups (10, 20 and 40 mg/kg/day) and one control group; another 10 young rats served as the normal group.

### 3.8. Step-Down Test

The step-down test was performed according to the previously reported method with some modifications [17], using an apparatus consisting of an acrylic box (25 × 25 × 25 cm) with a stainless-steel grid floor and a plastic platform (4.5 × 4.5 × 4.5 cm) fixed at the centre of the box. Electric shocks (36 V) were delivered to the grid floor for 6 s with an isolated pulse stimulator. First, rats were placed in the box to adapt for 3 min, then, electric shocks were delivered and the rats jumped on the platform to avoid the noxious stimulation. One day after the test was performed, and rats were placed on the platform. The latency to step down onto the grid for the first time and the number of errors when subjected to shocks with 5 min (the shocks were maintained for 5 min) were measured as learning performances.

### 3.9. Y-maze Test

The Y-maze test was conducted as previously described [17] using a Y-shaped maze with three identical arms at a 120° angle from each other (60 cm long ×16 cm wide × 32 cm high). The three arms were named arm A (start arm), arm B, and arm C. For the training, rats were placed inside the start arm while arm A and C were non-safe (shocks were administered); whereas arm B was safe (a safe zone was on the top of arm B). Then, a fixed resistance shock source was connected on an automatically operated switch and electric shocks (50 V) were applied. After shocks happened, the rats escaped from foot shocks by entering the top of arm B; this procedure was counted as one practice and the practice were repeated for 10 times. After one day, the rats were tested for 10 times, and the latency to enter the safe zone from the non-safe zone for the first time and number of errors displayed by entering the non-safe zone within 10 times were recorded as learning performances.

### 3.10. Measurement of AchE Activity

The rats were decapitated after being anaesthetized with sodium pentobarbital (40 mg·kg^−1^ i.p.), and skulls were split on an ice and salt mixture. The cerebral tissues were homogenized and the AChE activity in the cerebra concentration was determined by a automatic biochemical analyser using commercial kits.

### 3.11. Western Blotting for Determination of BACE1Expression

The rats were decapitated after being anaesthetized with sodium pentobarbital (40 mg·kg^−1^ i.p.), and skulls were split on an ice and salt mixture. Total hippocampus tissue proteins were extracted, and then equal amounts of protein (40 μg) were separated by sodium dodecyl sulfate/polyacrylamide gel electrophoresis (SDS/PAGE), blotted on polyvinylidene difluoride (PVDF), and probed with anti-BACE1 rabbit polyclonal IgG, and subsequently with goat anti-rabbit/HRP, and detected by chemiluminescence. To measure protein loading, antibodies directed against β-actin were used.

### 3.12. Statistical Analysis

All the results are expressed as the mean ± S.E.M. The statistical significance of differences was analyzed using SPSS software (SPSS for Windows 15.0, SPSS Inc., Chicago, IL, USA). The significance of the mean difference was determined by one-wayANOVA, followed by a LSD-t test for multi group comparisons. Probability values *p* < 0.05 were considered significant.

## 4. Conclusions

In conclusion, the administration of curculigoside can significantly enhance learning performance in aged rats. It also ameliorates memory deficits in aged rats by decreasing the activity of AchE in the cerebrum. In addition, the BACE1 expressions can be also down-regulated by treating with curculigoside, therefore, curculigoside may be a potent potential BACE1inhibitor that can be used for the treatment of AD in the future.

## References

[1] Um M.Y., Choi W.H., Aan J.Y., Kim S.R., Ha T.Y. (2006). Protective effect of *Polygonum*
*multiflorum* Thunb on amyloid β-peptide 25–35 induced cognitive deficits in mice. J. Ethnopharmacol..

[2] Yankner B.A. (1996). Mechanisms of neuronal degeneration in Alzheimer’s disease. Neuron.

[3] Wirths O., Multhaup G., Bayer T.A. (2004). A modified beta-amyloid hypothesis: intraneuronal accumulation of the beta-amyloid peptide-the first step of a fatal cascade. J. Neurochem..

[4] Liang D.P., Han G.C., Feng X.M., Sun J.Y., Duan Y., Lei H.X. (2012). Concerted perturbation observed in a hub network in Alzheimer’s disease. PLoS One.

[5] Lee H.G., Zhu X., Castellani R.J., Nunomura A., Perry G., Smith M.A. (2007). Amyloidbeta in Alzheimer disease: The null versus the alternate hypothesis. J. Pharmacol. Exp. Ther..

[6] Alzheimer’s Association (2009). 2009 Alzheimer’s disease facts and figures. Alzherimers Dement.

[7] Li X.L., Yang X.L., Cai Y.Q., Wang L., Wang Y.H., Huang Y.H., Wang X.X., Yan S., Wang L.P., Zhao X. (2011). Proanthocyanidins from Grape Seeds Modulate the NF-κB Signal Transduction Pathways in Rats with TNBS-Induced Ulcerative Colitis. Molecules.

[8] Peng W., Han T., Xin W.B., Zhang X.G., Zhang Q.Y., Jia M., Qin L.P. (2011). Comparative research of chemical constituents and bioactivities between petroleum ether extracts of the aerial part and the rhizome of *Atractylodes macrocephala*. Med. Chem. Res..

[9] Bafna A.R., Mishra S.H. (2006). Immunostimulatory effect of methanol extract of *Curculigo orchioides* on immunosuppressed mice. J.Ethnopharmacol..

[10] Vijayanarayana K., Rodrigues R.S., Chandrashekhar K.S., Subrahmanyama E.V.S. (2007). Evaluation of estrogenic activity of alcoholic extract of rhizomes of *Curculigo orchioides*. J.Ethnopharmacol..

[11] Venkatesh P., Mukherjee P.K., Kumar N.S., Nema N.K., Bandyopadhyay A., Fukui H. (2009). Mast cell stabilization and antihistaminic potentials of *Curculigo orchioides* rhizomes. J.Ethnopharmacol..

[12] Lakshmi V., Pandey K., Puri A., Saxena R.P., Saxena K.C. (2003). Immunostimulant principles from *Curculigo orchioides*. J.Ethnopharmacol..

[13] Wang Y.K., Hong Y.J., Wei M., Wu Y., Huang Z.Q., Chen R.Z., Chen H.Z. (2010). Curculigoside attenuates human umbilical vein endothelial cell injury induced by H_2_O_2_. J.Ethnopharmacol..

[14] Wu Q., Cheng X.W., Lei G.Q., Chen S.M., Chen J.K., Zhou T.S. (2007). Effect of Curculigoside on free radical scavenging. Chin. JMAP.

[15] Jiang W., Fu F., Tian J., Zhu H., Hou J. (2011). Curculigoside A ateenuates experimental cerebral ischemia injury *in vitro* and *vivo*. Neuron.

[16] Hampel H., Prvulovic D., Teipel S., Jessen F., Luckhaus C., Frolich L., Riepe M.W., Dodel R., Leyhe T., Bertram L. (2011). The future of Alzheimer’s disease: The next 10 years. Prog. Neurobiol..

[17] Zhang H., Han T., Yu C.H., Rahman K., Qin L.P., Peng C. (2007). Ameliorating effects of essential oil from Acori graminei rhizoma on learning and memory in aged rats and mice. J.Pharm. Pharmacol..

[18] Zarrindast M.R., Khalilzadeh A., Malekmohammadi N., Fazli-Tabaei S. (2006). Influence of morphine- or apomorphine-induced sensitization on histamine state-dependent learning in the step-down passive avoidance test. Behav. Brain Res..

[19] Green C., Shearer J., Ritchie C.W., Zajicek J.P. (2011). Model-Based Economic Evaluation in Alzheimer’s Disease: A Review of the Methods Available to Model Alzheimer’s Disease Progression. Value Health.

[20] Myhrer T. (2003). Neurotransmitter systems involved in learning and memory in the rat: A meta-analysis based on studies of four behavioral tasks. Brain Res. Rev..

[21] Himmelheber A.M., Sarter M., Bruno J.P. (2000). Increases in cortical acetylcholine release during sustained attention performance in rats. Brain Res. Cogn. Brain Res..

[22] Bartus R., Uehara Y. (1979). Physostigmine and recent memory: Effects in young and aged nonhuman primates. Science.

[23] Dawson G., Heyes C., Iversen S. (1992). Pharmacological mechanisms and animal models of cognition. Behav. Pharmacol..

[24] Song Y.T., Wang J.T. (2010). Overeview of Chinese research on senile dementia in mainland China. Ageing Res. Rev..

[25] Zhang Y.W., Xu H.X. (2007). Molecular and cellular mechanisms for Alzheimer’s disease: Understanding APP metabolism. Curr. Mol. Med..

[26] Kennedy M.E., Wang W.Y., Song L.X., Lee J.L., Zhang L.L., Wong G. (2003). Measuring human β-secretase (BACE1) activity using homogeneous time-resolved fluorescence. Anal. Biochem..

[27] Roberds S.L., Anderson J., Basi G., Bienkowski M.J., Branstetter D.G., Chen K.S., Freedman S., Frigon N.L., Games D., Hu K. (2001). BACE knockout mice are healthy despite lacking the primary beta-secretase activity in brain: Implications for Alzheimer’s disease therapeutics. Hum. Mol. Genet..

[28] Fu D.X., Lei G.Q., Cheng X.W., Chen J.K., Zhou T.S. (2004). Curculigoside C, a new phenolic glucoside from Rhizomes of Curculigo orchioides. Acta Bot. Sin..

